# Chiropractic as spine care: a model for the profession

**DOI:** 10.1186/1746-1340-13-9

**Published:** 2005-07-06

**Authors:** Craig F Nelson, Dana J Lawrence, John J Triano, Gert Bronfort, Stephen M Perle, R Douglas Metz, Kurt Hegetschweiler, Thomas LaBrot

**Affiliations:** 1 American Specialty Health 777 Front St. San Diego, CA 92101, USA; 2 Palmer Centre for Chiropractic Research, Palmer College of Chisopractic, 1000 Brady Street Davenport, IA 52803, USA; 3 Texas Back Institute 6020 W. Parker Road Plano, TX 75093, USA; 4 Northwestern Health Sciences University 2501 W. 84th St. Bloomington, MN 55431, USA; 5 University of Bridgeport 126 Park Avenue Bridgeport, CT 06604, USA

**Keywords:** Chiropractic, Evidence-Based Health Care, Health Care Professions, Professional Ethics

## Abstract

**Background:**

More than 100 years after its inception the chiropractic profession has failed to define itself in a way that is understandable, credible and scientifically coherent. This failure has prevented the profession from establishing its cultural authority over any specific domain of health care.

**Objective:**

To present a model for the chiropractic profession to establish cultural authority and increase market share of the public seeking chiropractic care.

**Discussion:**

The continued failure by the chiropractic profession to remedy this state of affairs will pose a distinct threat to the future viability of the profession. Three specific characteristics of the profession are identified as impediments to the creation of a credible definition of chiropractic: Departures from accepted standards of professional ethics; reliance upon obsolete principles of chiropractic philosophy; and the promotion of chiropractors as primary care providers. A chiropractic professional identity should be based on spinal care as the defining clinical purpose of chiropractic, chiropractic as an integrated part of the healthcare mainstream, the rigorous implementation of accepted standards of professional ethics, chiropractors as portal-of-entry providers, the acceptance and promotion of evidence-based health care, and a conservative clinical approach.

**Conclusion:**

This paper presents the spine care model as a means of developing chiropractic cultural authority and relevancy. The model is based on principles that would help integrate chiropractic care into the mainstream delivery system while still retaining self-identity for the profession.

## Background

It is always fashionable to speak of an issue or controversy as reaching a "crisis point," or of an organization or profession reaching a "crossroads" in its development. However such exhortations are often merely hyperbole. At the risk of committing this offense, we believe that the chiropractic profession today faces an exceptionally difficult set of challenges and, yes, a crisis. The nature of this crisis is the profession's continued inability to define itself. The chiropractic profession, more than 100 years after its founding, does not project a definition of itself that is consistent, coherent or defensible. The healthcare system is increasingly intolerant of such ambiguity and uncertainty; an intolerance which will only intensify in the future.

The primary purpose of this paper is to offer a coherent and defensible professional identity. We argue that chiropractic's identity is as a provider of spine care. We argue further that such a model is consistent with the best available scientific evidence, is consistent with the current public perception, provides benefit to both the profession and the public, and is capable of gaining for the profession the cultural authority it now lacks. In developing this model we established a set of criteria that the model must meet:

1. It must be consistent with accepted modes of scientific reasoning and knowledge.

2. It must accommodate future changes in scientific understanding.

3. It must represent a set of clinical competencies within the reach of practicing chiropractors.

4. It must be consistent, credible and communicable to external constituencies on whom the profession relies.

5. It must represent the evidence of practice experience.

6. It must find a substantial presence within the healthcare marketplace.

7. It must be compatible with the training, licensure, history and heritage of chiropractic.

## Part I: The Context of the Identity Crisis

### The Search for Cultural Authority

All healthcare disciplines have members who quibble over priorities and preferred belief systems. To prevent these squabbles from limiting advancement and productivity, there must be an understanding of common ground on which to build. With that in mind, it helps to ask "What are the core values/concerns held by the members of the chiropractic profession on which nearly all parties can agree?" We propose that there are a number of common factors even among the most diverse viewpoints within chiropractic.

• Patients benefit from chiropractic care.

• Over the past several decades, a substantial body of evidence has accumulated to inform decision-making on the value of chiropractic manipulation for low back, neck and headache complaints.

• A large population exists that is underserved by chiropractic.

• Extra-disciplinary competition is increasing, with greater encroachment on traditionally chiropractic domains.

• Significant barriers persist which obstruct the profession and its members from reaching their group and individual potentials.

With this common understanding we can ask, "Why is the modern evidence largely being ignored by policy makers and the access to chiropractic care being impeded by arbitrary obstacles?" To answer this question, we should step back and take a dispassionate assessment of how society invests its trust in professionals. The trivial answer identifies institutional bias as the cause; that is, policy makers rely solely on practitioners of medicine as their advisors. Although there is evidence that these attitudes are easing, stereotyping and bias toward the chiropractic profession remains pervasive. However, this is a superficial and inadequate explanation, as the sovereignty of medicine over healthcare has eroded significantly and its biases are increasingly evident to decision makers.

The more complete answer is based on the competition for cultural authority that each profession faces during its evolution. Cultural authority is granted by society based on recognition of a professional group's competency and legitimacy with respect to the domain over which it professes dominance. With cultural authority comes a certain degree of autonomy and privilege. Chiropractic has not anchored its cultural authority. Evidence of competency exists by virtue of years of practical experience and the presence of substantial evidence of effectiveness for methods of care for which the profession has held as its primary domain for the majority of the 20^th ^Century. It is on the front of legitimacy that we have failed. This failure is fueled by a mismatch between the profession's assessment of the value the practice of chiropractic offers and society's assessments of the same. Some chiropractors lament that the profession has done a poor job of educating the public about chiropractic. They posit that if we would just do enough advertising and more effective public relations, the resistance to using chiropractic services would decrease. As enticing as the argument sounds, that experiment actually has been done and has proven not only to be false but counterproductive. Canadian chiropractors found, in two separate samples, that marketing to the public about subluxation and the adjustment resulted in a backlash against the term "subluxation" and an increase in the public's desire to consult a medical doctor if they perceived they might have a subluxation [1]. The educational materials about chiropractic ideology were created by advertising professionals and broadcast under supervision of the chiropractors. The public is clearly not interested in, or receptive to this sort of message from the chiropractic profession.

Legitimacy, as defined above, is the active battleground today. Points of contention are the credibility of clinical claims for effectiveness of chiropractic manipulation for a variety of non-spinal conditions, cost of chiropractic care versus "standard care," and the presence of real or perceived unethical practices. Certainly, there is room to argue about most of these points. The profession is further encumbered by questionable institutionalized practices. For example, some practice consultants promote the policy of withholding administration of treatment on the first visit, preferring to reschedule the patient for a report of findings on a subsequent visit. Where is the clinical rationale for such practice? Are these doctors insufficiently skilled in interpreting the history and examination findings for a routine first visit without time to confer and study? Others promote the use of x-rays on nearly every patient in order to determine biomechanical deviations from a theoretical "model" of a normal spine implying that this information is so essential to successful treatment that the benefit outweighs the very real risk of radiation exposure [2]. These and other business practices promoted across the profession are tolerated without challenge by the rank and file. These practices degrade the credibility of the profession and its members as competent clinicians and diminish the public's trust and level of cultural authority. Considering these various threats to professional legitimacy, a new model is needed. Such a model will provide the chiropractic profession with common core values that permit the development and expansion of chiropractic as future evidence arises. A significant component of this new model must take into account accepted concepts of professional ethics.

### Professional Ethics and Chiropractic Identity

This discussion occurs within the context of chiropractic as a licensed healthcare profession. The status of "licensed healthcare profession" confers upon the chiropractic profession certain privileges, but it also imposes upon it a specific set of expectations and ethical obligations. Professional ethics differ from the ethics of mercantilism. For the customer, the relationship with a merchant has always been governed by the dictum *caveat emptor *or, let the buyer beware. Mercantilism demands that, for the merchant, pecuniary interests supersede others. Despite the fact that a chiropractic practice is typically a commercial, for-profit enterprise, the chiropractor is not governed by the dictates of mercantilism but rather by professionalism. Professions are so-called because they "profess" to have knowledge and skills beyond the comprehension of the laity. The theory of professionalism is predicated on this asymmetry of knowledge. Classically, the only professions were medicine, law, and the clergy, to which modern disciplines can be added, such as engineers, financial planners, etc. Hughes coined the expression *credat emptor*, let the buyer have faith, to describe the special relationship professionals have with their patient, client or parishioner [3]. Thus, chiropractors, as health professionals, are expected to make recommendations that are in the best interest of the patient, superseding the doctor's pecuniary interests.

As a result of patients' ignorance concerning the specialized knowledge of the professional, the faith a patient places in his or her doctor must extend to the information they are given by their doctor. The imbalance in knowledge means that the doctor not only must not lie to a patient (the ethical duty of veracity) but also must take pains to ensure that what they tell the patient is the truth (the ethical duty of fidelity), as best as it can be known by the doctor and understood by the patient.

At first glance, avoiding a lie and telling the truth may appear to be synonymous but they are not. If one honestly believes a piece of information told to another, then one is not lying. However, if that information is in fact not valid, one has not lied but has told an untruth. Thus, the person has erroneously transmitted incorrect information. Transmission of false information, if correct information is reasonably available to the profession, is a violation of one's duty of fidelity. The duty of fidelity is, in part, to comply with the reasonable expectations of the patient including the expectation that information given is in fact valid.

The ethics of professionalism require not only veracity, but also fidelity. Neither a chiropractor nor any other healthcare provider practicing under the protection of a licensed profession has the ethical right to promote unscientifically unreasonable beliefs. The principle of fidelity and the state of scientific knowledge regarding certain historical chiropractic beliefs should not allow the expression of these beliefs to the patient as clinical truths.

After D.D. Palmer founded chiropractic in 1895 his original body of work contained a number of postulates. Below, we will present an analysis of Palmer's Postulates. This analysis is not new and has been available to the whole profession. We do not regard this analysis as anything that should be regarded as controversial or contentious. It is merely an observation that conventional scientific methods should be applied to the principles of chiropractic. Despite the critical threats to the validity of this paradigm, a sizable proportion of the profession still holds these postulates to be valid [4]. The segment of the profession that continues to hold firmly to Palmer's Postulates do so only through a suspension of disbelief. Given that one of the philosophical pillars of science is skepticism, a suspension of disbelief or a lack of skepticism, is evidence of antiscientific thinking [5,6]. These stratagems to avoid the truth that Palmer's Postulates are unproven might be beneficial to the chiropractor, but are ethically suspect when they allow the practitioner to maintain a "faith, confidence and belief" in that paradigm to the patient's ultimate detriment.

### Misplaced Optimism

Over the past two decades it has been possible to view the chiropractic profession and its prospects for advancement in an extremely optimistic light. Compared to the profession's first 85 years of existence, the period of time from, say, 1980 to 2000 saw what seemed to be an unbroken string of successes. This period saw the ongoing development of the first chiropractic scientific journal, the first evidence (through clinical trials) of effectiveness of spinal manipulation, a legal anti-trust victory over the institution of medicine in the USA (*Wilk v. AMA); *an explosion in the number of students enrolled in chiropractic colleges, and the publication of a United States government report supporting the use of spinal manipulation for low back pain. In addition to these concrete developments the chiropractic profession benefited from the widely documented increase in interest and utilization of what has become known as complementary and alternative medicine (CAM) [7-9]. By the end of the century, as the result of these events and trends, the profession enjoyed a level of public acceptance (including that of other healthcare professions) that was unprecedented in its history. Some analysts of the healthcare system projected that by the year 2010 there would be over 100,000 chiropractors practicing in the United States alone [10,11]. It appears that reality will fall well short of that prediction.

As propitious as these developments appeared at the time, they have not secured the future of the chiropractic profession. A recent assessment by Richard Cooper MD, identified a variety of factors that threaten the future of chiropractic [12]. Dr. Cooper's analysis has captured the attention of many in the chiropractic profession and represents a realistic set of concerns, and calls for corrective action by the leadership of this profession.

During this same period, the healthcare system as a whole has undergone profound scientific, regulatory, political and economic changes that impose new expectations and responsibilities on all healthcare providers. An unprecedented level of professional accountability, predictability, and consistency are expected from all healthcare professionals. The chiropractic profession of the 21^st ^Century is obligated to provide a mature, ethical, and moral response as it seeks to anchor its professional jurisdiction and cultural authority.

### Internal Confusion

The chiropractic profession is not currently prepared to effectively meet these challenges. More than 100 years after its origins, the chiropractic profession remains focused on the internal debate "What is chiropractic?" – a quandary shared by many other stakeholders in the healthcare system. Perhaps as testimony to some underlying strength of chiropractic, the profession has managed to survive in spite of its confused self-vision. The more important issue is the profound organizational weakness suggested by the century-old debate on fundamental identity. It is difficult to fault decision-makers within the healthcare industry for any reluctance to embrace chiropractic when they do not know what it is they are asked to embrace.

There is a lack of uniformity and consensus within the profession about the proper role of chiropractic. Depending upon whose point of view is solicited; chiropractors are subluxation-correctors, primary care physicians (PCP), neuromusculoskeletal (NMS) specialists, wellness practitioners, or holistic health specialists. Within each of these models there are many competing factions. While the many professional subgroups of medicine (pediatrics versus cosmetic surgery, for example) converge, at least in theory, on broad but common ideology and professional attributes, the same is not true among the more divergent chiropractic factions. The differing chiropractic schools of thought form competing professional models that are not mutually compatible. Moreover, the disparities are indefensible in the context of the scientific, regulatory, political and economic criteria under which healthcare delivery is expected to operate. A number of models are impractical, implausible or even indefensible from a purely scientific point of view (e.g., subluxation-based healthcare), from a professional practice perspective (e.g., the primary care model), or simply from common sense (e.g. Innate Intelligence as an operational system for influencing health).

## Part II: The Failed Identities of Chiropractic

The "ACC Paradigm" document developed by the Association of Chiropractic Colleges in 1996 currently represents the closest thing to an official consensus of chiropractic identity [13]. This paradigm was formed by consensus among the 16 presidents of the member ACC institutions – a group generally believed to hold divergent beliefs and interests. We respectfully submit that this widely disseminated document does not fulfill the criteria outlined above. While perhaps a political triumph (getting all the presidents to sign on to the same document), it contributes little to the understanding of the profession's role in modern healthcare delivery by the relevant stakeholders. It is interesting that two major sources of contentious debate, the terms "subluxation" and "diagnosis," are both used in the same document. Even in that context, the reader may be left with a feeling of internal tension between them. It is otherwise a recitation of the trivial (the purpose of chiropractic is to optimize health), the obvious (doctors of chiropractic establish a doctor/patient relationship and utilize adjustive and other clinical procedures unique to the chiropractic discipline.), and of the tautological ([chiropractors]...employ the education, knowledge, diagnostic skill, and clinical judgment necessary to determine appropriate chiropractic care and management.) Experience with healthcare decision-makers at both the local and federal levels makes it appear highly unlikely that the ACC Paradigm will prove useful when these decision-makers assess the practical role of the profession.

The chiropractic profession has succeeded in a number of important ways. Foremost, it has provided an effective and much needed healthcare service; that is, the conservative management of common musculoskeletal disorders in a population of patients who would otherwise be less well treated. It has devoted its resources in creating a sizable infrastructure of schools, publications, research centers, and scientific conferences. It has succeeded in providing economically viable careers for tens of thousands of individual chiropractors. Inroads have been made in policy-making arenas and in efforts to train its members in practice protocols to facilitate a stronger interface with payers and policy makers. Interdisciplinary training has begun to establish a cadre of qualified clinical and fundamental scientists with a chiropractic background. Chiropractic has succeeded in transforming itself from a marginal discipline into one that has an opportunity (if it acts wisely) to become an integral part of the healthcare system.

The basic premise of this paper is that existing institutions within chiropractic have not expressed a model of chiropractic that empowers the granting of cultural authority, sustained economic viability, and scientific integrity. There are two particular perspectives we believe are at odds with the seven criteria outlined above: 1. The philosophical model and 2. The primary care model. In order to effectively make a case for the Spine Care model that we propose, we must first directly address these two differing points of view.

### The Philosophical Model of Chiropractic

The word "philosophy" is a much used but much misunderstood term within chiropractic. Most of the time those who invoke a "philosophical" argument are using the term in its colloquial sense: "I believe in a traditional set of chiropractic beliefs (chiropractic philosophy)." This set of beliefs is probably more correctly described as the ideology of chiropractic or the hypothesis of chiropractic, rather than as a philosophy.

This model of chiropractic has continued to advance a hypothetical model of health and disease divergent from other (particularly mainstream) modes of thought among the health professions. Indeed, some aspects of the hypothesis are now known to be at odds with scientific fact. To what extent can this chiropractic hypothesis be credited with the past successes of the profession? We argue that it is incorrect to interpret the success of the chiropractic profession as evidence of the validity of this chiropractic hypothesis. The profession has recorded limited successes in spite of what is largely the failure of this hypothesis.

#### What is the Chiropractic Hypothesis?

Before going further it is necessary to specify exactly what is meant by the *chiropractic hypothesis*. While there are an abundance and variety of competing versions of this hypothesis, all of which are ferociously defended by their adherents, it is still possible to identify several principles that are both common to the majority of these, and distinct from other healing systems. These principles are:

1. There is a fundamental and important relationship (mediated through the nervous system) between the spine and health.

2. Mechanical and functional disorders of the spine (subluxation) can degrade health.

3. Correction of the spinal disorders (adjustments) may bring about a restoration of health.

For the purpose of this discussion, these three principles will be referred to as Palmer's Postulates. There are a variety of different ways in which these postulates are expressed. The structure/function metaphor is often invoked – alterations of the body's structural components will result in functional aberrations and disease. Others emphasize the neurological aspect, the spine being both the source of noxious neurological stimuli and the locus of therapy where treatment can be administered to correct such stimuli.

But in the end, all of these modes of expression converge on essentially the same end point. That is the concept that the spine is not just another conglomeration of bone and muscle like the shoulder or the knee. Rather, it occupies a unique and privileged position in the makeup of the human body, representing both a vulnerability to our health and also a means of achieving optimal health. Expressions of Palmer's Postulates are ubiquitous within the profession and are not confined to extreme or narrow elements of the profession. These principles are to be found in some form in the mission statements of every North American chiropractic college and in the curricula of those colleges. They are further embodied in the ACC Paradigm paper. With the understanding that there is a great deal of room for qualification, clarification, and interpretation, we believe that Palmer's postulates do capture the essential hypothetical premise of chiropractic, and it is an error to underestimate the degree to which this theoretical model continues to define chiropractic. Even in the context of chiropractic research, where you might not expect a great deal of sympathy for these ideologies, Palmer's Postulates continue to guide the research priorities and agenda in the chiropractic profession.

We must also consider the concept of vitalism (in chiropractic, Innate Intelligence) as a component of Palmer's Postulates. Although there is a long historical legacy of vitalism, and although it continues to be a feature within many contemporary belief systems, there really can be no compromise on its inclusion as a defining principle of chiropractic. It was precisely the rejection of vitalism in the 18^th ^Century and the emerging understanding (through the invention of the microscope and other technological advances) of biological mechanisms that marks one of the watershed moments in the evolution of science. Chiropractic can choose to retain its vitalistic component only if it chooses to operate completely outside the scientific healthcare community. Vitalism does not require any further or more extensive analysis before rejecting it. To reject vitalism is to simply to announce that one accepts the conventional view of biology similar to the way one accepts the convention view of cosmology by rejecting a geocentric universe. In making this categorical rejection of vitalism one important distinction is necessary. While vitalism is incompatible with a valid professional model of chiropractic, it is not incompatible with an individual chiropractor's professional beliefs. An individual physician of any type may have religious convictions that inform their professional lives, and yet these convictions remain totally outside the domain of the professions' common identity. Similarly, an individual chiropractors belief (or non-belief) in vitalism can be considered to be entirely a personal matter so long as these beliefs do not distort the discharge of professional duties and obligations.

A distinction can be drawn between the "classical vitalism" described above and a "modern vitalism" that can be accommodated by conventional biomedical science. This modern vitalism is best described by the phrase *vis medicatrix naturae *– the healing power of nature. The truth of this proposition is indisputable. Nature, or more specifically, the body's natural healing mechanisms, is the principle mechanism by which any healing process occurs. Without these natural mechanisms (our immune system, our wound healing capacity, and countless other regulatory and corrective systems) life itself is barely possible.

This modern vitalism can also serve as a useful and valid guiding clinical principle. It implies, correctly, that these natural healing systems should be given every opportunity to operate with minimal interference by outside agencies, including by chiropractors. This sort of therapeutic minimalism is, in fact, an important part of model that we will propose.

We have asserted that Palmer's Postulates have failed. To understand our assertion, please first consider the nature of a scientific theory. A theory is an explanation. It is an effort to explain and make understandable a set of observations or facts that are otherwise confusing, paradoxical, or self-contradictory in some way, and for which our existing theoretical understanding offers an inadequate explanation. Implicitly, every theory is an answer to the question, "Why is it that...?" or, "How could it be that...?" A theory should be a solution to a puzzle. If a theory is sound it will solve the puzzle and also accurately predict as yet unobserved phenomena, thus increasing our ability to understand and manipulate our world. For example:

• William Jenner's theory of acquired immunity provides an explanation for the observation that milkmaids with cowpox scars do not contract smallpox.

• John Snow's theory of cholera transmission answers the question, "Why did almost everyone who drank from the Broad Street well contract cholera, and those who drank from other water sources did not?"

• Barry Marshall's theory of the infectious nature of ulcers answers, "Why does the occurrence of peptic ulcers, thought to be a psychogenic disease, very closely resemble that of infectious diseases?"

When looking at these and other successful theories, there are some important common elements:

• In each case, there was a riddle to be solved, a set of unexplained facts. The theories did not arise out of a vacuum. They arose out of the necessity to explain some new observations.

• The observations were accurate. The phenomena that Jenner, Snow, and Marshall were trying to explain were real. They had correctly perceived and recorded events in their world. For great scientists, observation implies a deliberate, systematic, and disciplined process, and not simply the casual perceptions of our surroundings and experiences.

• The observations could not be explained by existing theory. Each of the sets of observations described above were either at odds with our existing understanding of the world or simply not taken into account by other theories.

• All have survived repeated experimental test.

When one examines Palmer's Postulates in this light, their limitations become obvious. First, we need to ask what phenomena, exactly, are these postulates trying to explain? Particularly with respect to the first postulate that establishes the relationship between the spine and health, what observations gave rise to this hypothesis? Is there some set of facts or observations that cannot be understood without the insight provided by the postulates? D.D. Palmer might state that he was trying to explain why a deaf man with a vertebral misalignment recovered his hearing following re-alignment of that vertebra. However, there is no evidence that Palmer undertook any sort of systematic exploration of the spine/health relationship following his epiphany. What we know about D.D. Palmer suggests that patient and disciplined observation was not his forte. His method of discovery was by inspiration and revelation.

Subsequent generations of chiropractors might say that Palmer's Postulates are required to explain why there are so many healthy, happy, satisfied, apparently healed chiropractic patients. But there is nothing puzzling or mysterious about doctors having content patients – all healing systems from Ayurveda to chiropractic to medicine to therapeutic touch can make such claims. The power of natural history, regression to the mean, and non-specific treatment effects guarantee such results and unless one sets out to deliberately harm patients, it's difficult to avoid having satisfied and improved patients. Recovered patients are the inevitable consequence of having patients and no insight is gained into the validity of any of these healing systems by observing this fact.

The problem, simply, is that there is no need for Palmer's Postulates. There never has been a set of facts or phenomena concerning the relationship between the spine and health that require Palmer's postulates to understand them. The spine/health theory does not rest on any foundation of careful, comprehensive, and reliable observational data.

To illustrate this absence, the sort of observations that would require the explanations of Palmer's Postulates might look something like this:

• The observations that most persons with idiopathic scoliosis suffer from a wide range of diseases that non-scoliotics do not.

• The observation that persons with a specific spinal characteristic suffer inordinately from a particular health problem.

• The observation that back pain predictably results from certain postural defects.

The problem is that none of these observations, or any similar, are known to be true. Where evidence exists on these questions it points mostly in a direction the opposite of Palmer's Postulates. The real paradoxes and riddles are questions like, "Why is it that a scoliotic, osteophytic, degenerated spine with asymmetrical facets and collapsed discs can so often result in no clinical problems?" Or, conversely, why is it that someone with no identifiable anatomic spinal disorder can suffer from low-back disability. A disinterested party, dispassionately examining the evidence available today regarding the relationship between the spine and health, or the structure/function relationship, would arrive at the following conclusion:

*The human organism is highly resilient and broadly adaptable to a wide range of structural imperfections, and it is only after a rather high threshold of deformity is surpassed, that function is degraded*.

### The Primary Care Model of Chiropractic

The other great divide within chiropractic concerns the question of whether or not chiropractic is a primary care profession. Unfortunately, just as the word "philosophy" is routinely misused, so is the concept of "primary care." Paradoxically, even the extremes of the profession on the philosophy question (e.g., Sherman College and National University) both endorse the notion of chiropractic as a primary care profession. This agreement does not suggest that chiropractic, as primary care is a valid and compelling concept. Rather, it suggests that the concept has been unexamined and hastily adopted. This section will examine the meaning of primary care as it applies to chiropractic.

#### What is Primary Care?

There are several definitions of primary care physicians (PCP), but possibly the most accepted is the definition provided by the Institute of Medicine in a 1996 report. It defines primary care as, "the provision of integrated, accessible, health care services by clinicians who are accountable for addressing a large majority of personal health care needs, developing a sustained partnership with patients, and practicing in the context of the family and the community [14]." The essence of the IOM definition, as well as others, is of a primary care physician as a generalist and not a specialist. This is most easily illustrated by the prototypical examples of PCPs as identified in the IOM report: family practitioners, pediatricians and internists. The report also identifies nurse practitioners and physician assistants who are specifically trained in providing primary care.

In each of these examples, the PCP provider sees a wide range of complaints (respiratory, cardiovascular, gastrointestinal, and musculoskeletal) within the specified patient population, treats most of these complaints directly, and refers the rest as appropriate. Even in the more limited primary care professions (nurse practitioner, physician assistant) the generalist theme is also fundamental to defining their practice. These practitioners provide more limited care than medical PCPs and act more in a triage capacity than in a therapeutic capacity depending on complexity of the case. But there is general agreement that these providers fit the primary care model when they opt for the generalist practice.

To what extent do chiropractors satisfy the generalist model? Not at all, as it turns out. The most obvious index of this is the chiropractic patient population. In the last decade there have been many studies, surveys, and analyses that have described and characterized the chiropractic patient population [15-21]. These studies all reach the same conclusion: the chiropractic patient population consists, almost in its entirety, of persons with musculoskeletal pain complaints, the overwhelming majority of which are spine related. A small subset, approximately 5%, of patients have headache as a primary complaint. Any reasonable estimate would place the percentage of chiropractic patients with somatic pain at >95%. Most of the balance of patients receive some sort of "maintenance" or "wellness" care. A very small number (<1%) have complaints that fall outside these categories.

It might be argued that the make-up of chiropractic patient population simply represents a cultural and historical artifact; that the public has not been educated as to the suitability of chiropractors as PCPs and it's simply a question of providing proper education to the public on this matter. The fundamental limitations imposed by the profession upon itself make this argument implausible.

The first limitation is therapeutic. By intent, chiropractic has limited its therapeutic armamentarium to manual and physical techniques. This limited set of therapies is well suited to the set of complaints normally seen by and successfully treated by chiropractors. This limited set of therapies also offers the advantage of a very low risk of harm. However, this limited set of effective services is poorly suited for providing primary care. Beyond musculoskeletal conditions, there are very few conditions for which manual therapies provide optimal effectiveness. The vast majority of human health problems that require an intervention do not fall within the chiropractic therapeutic spectrum. Chiropractic cannot simultaneously retain its limited set of therapies and pursue primary care status.

It might be argued that even with its therapeutic limitations chiropractic could provide the services of a diagnostic generalist and make therapeutic referrals as needed. However, the defining characteristic of any diagnostic generalist is a rigorous training and experience with the spectrum of disorders likely to be encountered. Any intellectually honest analysis of this question will not support the supposition that chiropractic training provides such rigor in this domain. The length, breadth, and depth of chiropractic clinical training do not support the claim of broad diagnostic competency required of a PCP. Studies of chiropractic intern clinical experience provides no evidence that chiropractors are trained to a level of a diagnostic generalist for non-musculoskeletal conditions [22,23]. For chiropractors to describe themselves as PCP diagnosticians is to invite comparisons to other PC diagnosticians, i.e., family practitioners, pediatricians and internists. Such comparisons will not reflect favorably on chiropractic.

Finally, it might be argued that although the chiropractic profession is not currently trained to provide PCP care, it *could *be and we should set ourselves to the goal of making this happen. If a chiropractor as PCP is not at this moment a reality, we can imagine a different reality in the future in which the Chiropractor/PCP model made sense. What would have to change for this reality to come true? At a minimum, the following:

1. Chiropractic would have to dramatically increase the length, breadth and depth of its clinical education at all its accredited institutions.

2. Chiropractic would have to develop an acceptable solution to its therapeutic limitations, either through changes in state licensure or by some as yet unidentified process.

3. Chiropractic would have to demonstrate its ability to deliver safe and effective care beyond its current model.

4. Having achieved goals 1-3, the chiropractic profession would have to change the view of the public and other health professions of chiropractors as back doctors.

5. And finally, the profession would have to convince the healthcare marketplace (in which there is no current or anticipated shortage of PCPs) that there is some point to expanding the number of PCPs.

These events do not appear to be likely to occur in the near future.

## Part III: The Spine Care Model

In the course of discussions among the authors of this paper as well as others who were involved in the process, it became clear that there were many points of consensus. These consensus points are listed below in the approximate order of their importance to the model.

• Chiropractic as an NMS specialty, with particular emphasis on the spine.

• Chiropractic as a portal of entry (POE) physician/provider.

• Chiropractic as a willing and contributing part of the evidence based healthcare (EBHC) movement.

• Chiropractic as conservative/minimalist healthcare provider.

• Chiropractic as a fully integrated part of the healthcare system, rather than as an alternative and competing healthcare system.

Incorporating all of the above elements, chiropractic should actively market itself to the public and to the rest of the healthcare system in a sober and moderate fashion, and with a message that is completely compatible with current social, economic, political, and scientific realities. The balance of this paper will be devoted to examining these issues.

### The Dental Model

As a start to defining the model it is helpful to find another profession with analogous clinical jurisdiction e.g. focused practice emphasis on a region or set of problems, limited therapeutic regimen, and broad public identification with a selected role in healthcare. We believe the dental profession is a practical and successful parallel. Consider the advantages of the dental model:

• Dentists and dental surgeons have established themselves as the absolute, undisputed authorities in tooth care, a critical and essential component of human health, and a contributor to care for orofacial disorders generally. No one suggests they should not be portal of entry providers. No other profession considers usurping the role as tooth-care expert.

• In the public's perception, dentists are among the most highly esteemed of the healthcare professions.

• Dentists are recognised with the title "doctor" and reap the social, professional and financial benefits of their reputation and training.

• Dentists, though primarily focused on the dental anatomy and disease, are also expected to understand differential diagnosis of conditions related to their area of focus.

• The services that dentists provide, focused though they are to tooth, gums, and mouth, are of immense benefit to the health and well being of the public.

As this model unfolds, this is the image we might want to keep in mind – chiropractors as dentists of the back.

### The Vocational Role of Chiropractic: Treatment of Back Pain

The purpose of this essay is to define chiropractic as a *profession*. The term is emphasized because it is necessary to remind ourselves what this means and what are the consequences of being a profession. A profession is not defined by a set of ideas and values. Professions may have ideas and values, but these are not what distinguish or differentiate them as professions. Those organizations that are defined by ideas and values are entities like political parties, ideologies, religions, or organizations devoted to narrow issues like pro-life or pro-choice organizations. For such organizations, it is correct to state that the idea comes first, and everything else – strategy, tactics, etc. – flows from the question: what will best promote our idea?

A profession is about a specific vocational role that the profession fills. A profession is defined by the work it does and the role it fills, not by its ideas and values [24]. The ideas and values of a profession must be secondary – they exist to answer the question: "How can we best discharge our designated role in society?" Professions do not or should not exist to be champions of ideas. This is most specifically true of the licensed professions. Society grants a license, a franchise, to a profession, not so that profession can champion its ideals, but because society wants some specific work done and it feels that granting a franchise is the best way to do it. This social contract is quite explicit. In most cases the vocational role of professions is quite obvious and can be stated in a few syllables:

• Tooth and gum care.

• Design and engineering of buildings.

• Measurement of financial performance.

• Legal services.

This simple and coherent vocational role is what the chiropractic profession seems to have so much difficulty in defining, and what the ACC paradigm fails to provide. Among the reasons for this failure is that chiropractic has always been confused about the concept of a profession and has tended to view itself a champion of ideas rather than as a provider of service. This confusion is perhaps understandable in an historical context. Chiropractic didn't begin as a profession; it began as an idea or set of ideas (vitalism, subluxation). Palmer and company were champions of these ideas, competing with charlatans and learned (not scientific) professional rivals for status. Over the decades, the institutions and each individual chiropractor saw themselves as a champion of the chiropractic idea.

But, at some point over the last 100 years, and unbeknownst to the individuals and institutions of chiropractic, it became a profession with a specific vocational role. As these thousands of chiropractors over the decades were advancing the ideals of the profession through manipulation of the spine, the public, which is largely disinterested in the ideas, decided that chiropractic had a professional role to fill. Thus, creating the profession as it exists today.

The irony is that the specific professional/vocational role that chiropractic fills is obvious to the majority of patients and other non-chiropractors – it is chiropractors themselves who seem to be confused by the issue and who then provide confounding answers and contradictory testimony to policy makers. For all other mainstream healthcare professions it is easy to provide a straightforward answer to this question of role. Whether it is an optometrist, a pediatrician, a dentist, a family medical practitioner, or a psychologist, each has clinical domain that is essentially self-evident. For all other PCPs, and POE (point of entry) providers there is a relatively clearly defined patient population for whom the practitioner is an appropriate provider. This patient population may be defined by age, gender, and most importantly, by nature of healthcare problem or complaint. There may be some disagreement among various professions at the margins of this question, but only at the margins.

A somewhat different state of affairs obtains for those health professionals whose clinical purpose is not defined by a patient population, but by a specific technique or skill. For example, consider a general surgeon, pathologist or radiologist. The potential patient population of these providers is virtually everyone, as a function of their specific need for the service. To some this might seem an attractive model for chiropractic – our patient population is everyone who needs spinal correction, which is to say, everyone. In fact chiropractic has attempted this by defining itself in metaphysical terms (Innate Intelligence), as a technique (chiropractic adjustment), and as an ideology (Palmer's Postulates), rather than as a provider of specific clinical services. The failure of this approach is in fact the genesis of this paper. To define the clinical purpose of chiropractic, it is necessary only to observe what chiropractors actually do and for what purposes patients seek care from doctors of chiropractic: the provision of portal-of-entry care for the diagnosis and management of back pain, neck pain, and related disorders. In the shorthand that the public might use, chiropractors are back doctors. Restating some of the earlier points, this conclusion is based on these facts:

• The population – Over 90% of chiropractic patients seek care for back-related problems.

• The evidence – Clinical science provides a body of evidence for the effectiveness of chiropractic care for back pain, neck pain, and headache.

• The education and training – Chiropractic clinical education and training are focused almost exclusively on the conservative treatment of spine complaints.

• The public identity – The public perception of chiropractic is that of a back pain specialist and nearly a total rejection of an alternate role.

• The competition – The legitimate professional claim for chiropractic in the remainder of healthcare and public policy lies strictly within the domain of back- related pain outside the bounds of medical emergency.

• The claim of professional jurisdiction – Credibility for the claim, either diagnostically or therapeutically, for a broader role beyond the realm of this definition is lacking.

Should the chiropractic profession concern itself with what others think? It should, must and had certainly better do so as it is reliant upon its consumers for its existence. A profession *is *a public trust. The privileges accorded to a member of a profession are in direct exchange for professional members' service to the public. It is nonsensical to organize a profession in terms that are at odds with the public's perceptions of its interests unless a compelling and persuasive argument can be made that the public's perception is not in their best interest and is amenable to change. We maintain that there is no such argument. In fact, efforts to launch such a campaign have failed. For example, two recent public relations efforts have been attempted by chiropractic organizations. These efforts were preceded and followed by measure of the public attitudes toward the profession. In both cases, efforts to convince healthcare consumers about the role of subluxation in their lives backfired miserably. Not only were few persons encouraged to consult a chiropractor, but, the number of skeptics was increased and more respondents stated that they would seek a medical consultation first following the PR effort than before the campaign. The argument that the public can be persuaded to understand and accept the subluxation model of chiropractic has been tested and it has failed.

Finding a substantial presence within the healthcare marketplace is well satisfied by the spinal care model. A recent analysis of healthcare and productivity costs associated with specific complaints reveals the following:[25]

• Three of the top 10 conditions suffered by the US population (in terms of costs) are back pain related.

• Collectively, the annual rate of back pain-related healthcare episodes is 157 episodes per 1000 covered lives, making it the single most common complaint.

• Collectively, the annual direct healthcare cost for back pain is US $122 per person, second only to the cost of managing angina pectoris.

• Collectively the annual average cost of payment for lost work and short-term disability is US $87 per person, making back pain the most costly of all diagnostic categories in disability-related costs.

It should be noted that while some of these back pain episodes are undoubtedly not chiropractic cases (that is, they are legitimate in-patient or surgical cases) almost all are. Conservatively, at least 75% of this spine care patients potentially stand to benefit from chiropractic care, compared to the 12-17% who currently avail themselves of the services. This study, and many others, provides ample evidence that the clinical domain of back pain provides an enormous potential patient base and subsequent economic base for chiropractic.

Thus, the logic of the chiropractor as spinal care doctor proceeds as follows. First, chiropractors are *de facto *back pain/spine doctors seeing a limited proportion of the population, today. That is, as chiropractic is currently practiced (even given the confused message that chiropractic projects) it is entirely dependent on back pain/spine care for its economic survival. Second, the back pain market is enormous and can provide, by itself, a sufficient patient base to support the entire profession. Third, expansion of the chiropractic market share for spine-related symptoms is hindered primarily by a lack of credibility of its claims and the resistance that this lack generates among consumers and policy makers. Fourth, chiropractic has the most clinical training, expertise, and demonstrated clinical effectiveness as conservative back pain/spine doctors. Fifth, chiropractic as a spinal care specialty is the only basis on which the profession is understood and accepted by those outside the profession. Sixth, there is nothing to be lost, either in the short or long term by adopting this strategy. The state of mind regarding the profession that we would like to make is: *Go to a DC for your spinal health and prevention as you would go to your dentist for your dental health and prevention*. We reemphasize that there is nothing to be lost, either in the short or long term, by adopting this strategy. This model of chiropractic as the spinal care profession is in no way intended to preclude the patient population of extra-spinal musculoskeletal complaints. However there are several reasons why we feel it is reasonable to de-emphasize, relative to spinal care, this patient population.

1. It represents a very small percentage (<5%) of the current chiropractic patient population.

2. There is very little evidence of effectiveness of chiropractic care for this population and it is unlikely that a sufficient number of these patients present for care in order to conduct appropriate studies in a reasonable and timely manner.

3. It is unclear what advantage(s) chiropractic care might offer relative to other providers (physical therapists, rheumatologists, etc.) for care of these problems.

4. It is likely, with today's knowledge, that the proportion of extra-spinal MS patients for whom conservative manual therapy is the optimal approach is significantly less than is the case for spinal conditions.

5. There is far less public awareness or willingness (as reflected the utilization of services) of chiropractic as a provider of care for these conditions.

### Portal of Entry Status

We suspect that among some chiropractors there is confusion about the two terms "primary care," and "portal of entry," and that this confusion is at least partially responsible for the enthusiasm for the primary care model. The American Chiropractic Association, in fact, uses both terms to describe the profession [26,27]. However, primary care, as discussed above, describes a generalist provider, while a portal of entry (POE) describes a health care provider who may practice autonomously and to whom the public has direct access. The confusion lies in the belief that in order to achieve portal of entry status one must first be a primary care provider. A primary care physician is certainly a portal of entry provider, but one need not be the former to be the latter. The examples of dentistry, optometry, and clinical psychology illustrate this point.

On this question there is virtual unanimity in the chiropractic profession and the logic of chiropractors as portal of entry providers is obvious to all but the most vociferous opponents of the profession. The POE status of chiropractic is guaranteed in all the 50 American states as well as in most countries outside the US where chiropractic is licensed. There is no credible case that can be made that in some manner the public will be better served by requiring them to go through a gatekeeper (presumably an MD) to seek care from a chiropractor. The primary impediment to full implementation of portal of entry status is not a regulatory or a statutory problem, but a problem of inter-professional trust. Within specific health care delivery and financing systems there are gatekeeper provisions that require patients to be referred for chiropractic care. These gatekeeper arrangements arise either through concern of improper diagnostic workup and clinical decision-making, or through concerns of utilization abuse. While the fairness and appropriateness of these gatekeeper requirements is certainly in doubt, the surest way for the profession to protect and expand its POE status is to establish the cultural authority, and thus, the trust, that will make these gatekeeper provisions unthinkable.

### The Acceptance of Evidence-Based Healthcare

Fifteen years ago, the editor of the *New England Journal of Medicine*, Arnold Relman, MD, wrote an editorial in which he announced that healthcare had entered a new age, The Age of Accountability [28]. What he was describing is what we now call Evidence-Based Healthcare (EBHC). During the same period of time in which the CAM revolution was taking place, a second less visible revolution was also taking place – the establishment and application of the principles of EBHC.

Evidence-based healthcare is often ill defined, misunderstood, and a basis for concern or even fear by health-care providers. One of the best definitions we have seen appeared in an editorial in the *British Medical Journal *in 1996 written by some of the most prominent educators in EBHC, David Sackett and his colleagues [29]. They defined EBHC as the conscientious, explicit and judicious use of current best external scientific evidence in making decisions about the care of patients. EBHC does not mean that individual clinical experience is of limited or no value; on the contrary, EBHC offers advice on how to maximize the clinical expertise and combine that with the best available external scientific evidence that usually comes from systematic reviews and evidence-based clinical guidelines. Another important aspect of EBHC is the identification and incorporation of informed patient preferences.

The concern and fear that many health-care providers have is that EBHC will be misused by healthcare policymakers and health insurance companies to curtail the cost and limit reimbursements. Such policies would be inconsistent with the fundamental principles of EBHC. Clinicians who practice EBHC will develop the skills to identify and apply one or a combination of the most efficacious treatments, which if based on the individual patient profile will tend to maximize the benefit and minimize the risk. This may sometimes raise rather than lower the cost of their care. EBHC is not about proof or certainty. It is a method of dealing with uncertainty. It is about weighing the evidence and weighing alternatives.

There is one additional element of EBHC that requires amplification. It is important to understand EBHC does not mean care should be withheld if there is no proof of efficacy from systematic reviews or meta-analyses of randomized clinical trials. Absence of evidence of treatment efficacy does not equate with evidence of its absence. Such a standard would produce therapeutic paralysis. For example, there are virtually no clinical studies, chiropractic or otherwise, that have evaluated the effectiveness of treatment for thoracic spine pain. Obviously it is not reasonable to send a thoracic spine patient home with the apology, "Sorry, can't treat you – no evidence of efficacy." It is however essential that clinicians understand that evidence ranges from the weakest (clinical experience or expert opinion) to the strongest (high quality systematic reviews of all available relevant scientific studies). Many different systems for grading the evidence and making recommendations currently exist, and major efforts are underway internationally to standardize this process.

### The Role of Clinical Experience in EBHC

The central premise of EBHC is that even the most well thought out, tightly reasoned, and scientifically plausible treatment regimen may not produce benefit to the patient. The scientific literature is overflowing with examples of commonly used treatment procedures or regimens which were based on sound pathophysiologic principles, but were ultimately found to be of no benefit or even harmful to the patient [30-33]. For the chiropractic profession the lesson is obvious. Whether its Palmer's Postulates or any of its innumerable variations (in the form of proprietary techniques) the chiropractic profession cannot predicate its clinical validity upon untested theories.

EBHC principles state that healthcare providers need to combine their clinical expertise with the best available external evidence and that neither alone is sufficient. The most difficult and counter-intuitive notion for clinicians to accept is that their everyday experience of satisfied and seemingly recovered patients is not evidence of clinical effectiveness. There are several competing explanations for this apparent success. Many of the conditions treated by chiropractors, such as back pain, neck pain and headache, have a self-limiting natural history although they may be recurrent. The nonspecific placebo effect of the doctor-patient relationship explains many of the results attributed to specific interventions. Clinicians notoriously have selective memories and tend to recall success stories and generalize from those. The lack of systematic and standardized recording of diagnoses and clinical outcomes that could be gathered in databases and summarized objectively prevents clinicians from having an unbiased knowledge of the effect of their therapeutic efforts. EBHC recognizes the limitations and inherent unreliability of uncontrolled clinical observations and impressions and the inevitability of mistaken conclusions based on those uncontrolled observations. EBHC stresses the importance of outcomes-based clinical research, of regularly consulting the scientific literature, of optimizing the clinical skills of healthcare providers, and taking patients preferences into account.

As a practical matter, many chiropractors, and medical physicians as well, fear that EBHC will result in a change and possible limitation of their individual clinical prerogatives. They are correct in this conclusion. It is in fact the precise purpose of EBHC to help define what constitutes best practices-different from what would be the case if individual providers were given free reign to continue with their habitual practice behavior based exclusively on clinical experience.

It is also important to recognize that EBHC is in its infancy. The processes of EBHC will continue to accelerate in the future. When there is enough evidence to justify it, relative to a particular condition, we see the development of "disease management" programs. Disease management represents nothing more than a highly evolved implementation of EBHC. When there is sufficient evidence available, it becomes possible to implement very specifically defined (and also, very effective) treatment protocols that take into account important differences in prognostic factors among patients. These programs already exist for congestive heart failure, asthma, urinary tract infections, diabetes and other common illnesses. It is currently not feasible but only a matter of time before disease management of back pain, for example, becomes possible and necessary. If the chiropractic profession hopes to make progress within the healthcare mainstream, it must go out of its way to be clear that it understands EBHC, that it embraces its principles, and that it is acting to advance its implementation.

### Conservative/Minimalist Healthcare

One of the general truths revealed through the application of EBHC is that less is often more in healthcare. There are countless examples where clinical studies have shown that providing less healthcare, doing fewer procedures, taking a more conservative approach, even doing nothing, is superior to a more aggressive approach. This idea has always been understood at some level (it is the premise behind the "First, do no harm," doctrine), but it has been difficult for our healthcare system to act on the idea. Most incentives, economic and otherwise, propel care in the direction of more, rather than of less.

Chiropractic has a considerable advantage when it comes to implementing the doctrine of "First, do no harm." The scientific literature strongly supports the finding that chiropractic, and specifically, spinal manipulation, is generally safe. The evidence regarding spinal manipulation indicates that the incidence of serious injury is, if not trivial, extremely low. Of the more common adverse effects resulting from spinal manipulation, nearly all are transient and minor. Overall, the safety profile of spinal manipulation is excellent and more so when compared to other treatment options.

Through historical precedent, by intent and by design chiropractic has evolved using a conservative therapeutic regimen consisting of manual and physical therapies as well as exercise. The clinical effectiveness of this approach has been established, the safety profile is excellent and there are distinct cost advantages to this approach when used appropriately. We see no reason to change the therapeutic scope of chiropractic. It should be understood that this is a contingent position. It is contingent upon the continued clinical effectiveness and superior safety profile of these conservative modalities relative to other more aggressive interventions, particularly medication and surgery. None of these therapies, conservative or otherwise, will remain static and as they are improved upon in the future their relative merits may change as well. Chiropractic's allegiance to a conservative therapeutic regimen is valid only as long as it remains a clinically and economically sensible thing to do.

In order to fully exploit the advantages of its current conservative approach the chiropractic profession must take active measures to curb abuses that run counter to this approach. There is a long tradition in the profession of promoting the idea that the unadjusted spine is an invitation to disease. There are practice management procedures that attempt to maximize the number of patient visits that can be extracted from each new patient. There is nothing conservative about a treatment regimen of 3-times-a-week, forever. There is a commonly expressed notion among the public and among other health professionals that chiropractic treatment is open-ended and often never-ending. By these and other similar offenses, chiropractic has surrendered the high ground when it comes to delivering conservative healthcare. Using its current set of conservative therapies and incorporating the best published data, chiropractic can make a credible case that it offers the best combination of safety, effectiveness and cost for the management of back pain.

### Integration

The spine care model will facilitate integration of the chiropractic profession into the mainstream of healthcare. Integration offers substantial advantages toward addressing professional values and resolving the concerns outlined in the beginning of this essay. It is the primary vehicle by which cultural authority can be anchored for its competencies currently supported in the scientific literature. Integration brings with it a greater responsibility, but also brings the resources and patient access necessary to answer the core issues common to all chiropractic ideological debates.

Chiropractic has operated a parallel tract of professionalization since its inception. As Abbott observed, parallel development is associated with significantly greater obstacles and opposition than a profession that evolves as a branch from common roots [24]. While there is much accomplishment to appreciate, the profession continues to be hindered by limited resources for its stability and advancement. While at least partial acceptance and licensure has been achieved in many countries, many challenges remain before the profession can establish its reputation of competence and legitimacy necessary for full cultural authority. In modern society, training and licensure is no longer sufficient to demonstrate competence. That requires continued validation, which, in turn, requires credible data and a coherent identity. Legitimacy is eroded if practice patterns are tied to reimbursement, profit margin, or professional rivalry.

Perhaps the most fundamental question that the chiropractic profession must answer to finalize its cultural authority is: "Does the chiropractic profession continue to position itself in opposition to orthodox medicine, or does it stand as an advocate of the patient's best interests, as a part of mainstream healthcare, along with medicine?" To date, the chiropractic profession has enjoyed the ability to evade that decision, occupying an ambiguous position between opposition of medicine and full participation in the mainstream. The profession and its members have often used marketing methods offering an alternative to medicine. At the same time, political activism in the USA has yielded many of the benefits of the mainstream through participation in the private third-party payment system, in Medicare and a variety of other state-sponsored programs, as well as inclusion in student loan programs and in the Veterans Administration and Defense Department programs.

The emergence of the phenomenon of CAM has also played a role over the past few decades. Analyzed in both scientific publications and the popular media, the CAM phenomenon is now a well-established and positively recognized element within our healthcare system. The difficulty is that the CAM phenomenon has reinforced the cultural authority chasm in which the profession finds itself. There is such significant evidence supporting chiropractic benefits for spine care that it is considered by policy makers to be more mainstream than CAM. Yet, professional claims over the non-musculoskeletal domain and questionable practice behaviors obstruct full consideration within the mainstream by purchasers of healthcare research and delivery. While for some, the notion of being an alternative healthcare provider has a certain cache; this notion is neither clinically nor scientifically justified. It is a cultural and political status crafted by society for the prime purpose of evaluating whether the claims made by such practitioners are of any value. In the long run, the evaluation will elevate some and will degrade others. As noted by Marcia Angel, in the special *New England Journal of Medicine *issue on alternative healthcare: *"There is only medicine that has been adequately tested and medicine that has not, medicine that works and medicine that may or may not work."*[34].

Further, the barrier to entry into CAM is too low for the profession of chiropractic. There are too many CAM-related procedures, practices and providers that lack scientific rigor. Chiropractic is by far the most mature profession among those associated with CAM. Its pre-professional requirements are the highest; the professional education the most developed; its research capacity, the most advanced; and its presence in the healthcare marketplace, the most comprehensive. Simply, the chiropractic profession undermines its legitimacy and authority by striving to remain within the CAM phenomenon.

We do not believe that this intermediate status of half-alternative/half-integrated is sustainable for much longer. The profession needs to decide in which of these two camps to plant both feet. Without the intent of its members, like the question of chiropractic's clinical purpose, this question of integration has largely been decided by default – chiropractic is an integrated part of the healthcare system, and the profession must continue to promote further integration. The benefits of integration to the profession are too great to ignore. To be a part of the system is to have access to all the resources of the system- funds for research, state supported education institutions, training opportunities in hospitals and other integrative clinical settings, access to other educational institutions and nearly universal inclusion in all reimbursement systems. We must take particular note of the recent approval and funding of a chiropractic college at Florida State University. This was a tremendously important development in this history of chiropractic and one that had the potential to profoundly deepen and accelerate the integration of chiropractic into the mainstream. What is unsettling is the fact that the college failed before it could even enroll a single student almost exclusively because of the failure of the profession to advance a coherent credible message regarding its role within the healthcare system.

For the profession, integration will insist on clinical accountability and responsibility, a demand that our members feel even now with the increased pressures of healthcare reform. The rewards of integration, however, are extensive. The experience of individuals who have broken down many of the barriers and succeeded in establishing chiropractic programs within mainstream healthcare centers is expanding. The development of chiropractic facilities for the United States Congress, within the military, and within private musculoskeletal centers has been universally positive for patients and for the participating chiropractors. Beside personal professional success, these experimental programs have bought additional trust and credibility within the system. The participants have experienced a hitherto unheard of expansion in clinical exposure. Increased patient volumes, case variation and complexity and provider satisfaction are evident. Doctors can experience a new freedom from the tyranny of personality cults and practice-builder manipulations. New opportunities for career track development are opening as healthcare policy makers, clinical and basic scientists and educators for interested individuals who are interested in cross training.

For the profession's infrastructure, integration confers enormous advantages. By functioning within the mainstream of healthcare, chiropractic will be able to gain access to a far broader population of patients and practice within a more varied set of patient care settings. The academic institutions will be able to free themselves from the stranglehold of economic dependence on tuition and the political reliance on ideological gurus who manipulate alumni and support to garner institutional control. The results will expand the jurisdiction and influence of the profession's cultural authority as warranted. The profession will be a member at the table of discussions and debate over the future of healthcare delivery. As a participant, chiropractic autonomy over its domain will be more certainly assured than in our current reactive conflict postures.

### Other Issues

There are a variety of other questions that bear upon the issue of the chiropractic model.

1. The role of spinal manipulation in chiropractic. There is no foreseeable future in which spinal manipulation is not the primary therapeutic tool of chiropractic. But if or when that changes, it will be a function of the progress and evolution of clinical science, and not as a principle of chiropractic. That is, SMT should be viewed not as a defining element of chiropractic, but simply as what we happen to do. Invoking the dental analogy again, dentists do not define themselves as "implanters of dental amalgam," although that is probably what they do the most. As the discussion above on chiropractic philosophy illustrates, to do otherwise, to focus exclusively on SMT, as *the *chiropractic therapy will hinder our ability to pursue a more optimal treatment for back pain. We must make sure that we are prepared and equipped to identify and deliver whatever conservative therapies for back pain prove to be most effective.

2. The use of drugs. Should chiropractors seek limited prescribing rights as has been attempted in the past? Or should chiropractic promote itself as a "drugless" profession? We believe the answer to both these questions is "no." In the first instance (should chiropractors prescribe), clinical science has created a very strong case for conservative healthcare. Much of the advantage that chiropractic currently enjoys in the realm of back pain treatment (in terms of cost, safety, and satisfaction) is directly attributable to its conservative (non-drug) interventions. The US osteopathic experience is informative in this regard. Given the option of prescribing and using other more invasive interventions, it is much easier to prescribe than it is to use a manual therapy, and the role of manipulative therapy has diminished and nearly vanished from the profession.

Regarding the second question (should chiropractic promote its "drugless" nature), we should not promote the juvenile notion that drugs are bad and SMT is good. Our non-use of drugs should simply be regarded as a conscious decision to focus on a particular therapeutic approach, rather than a comprehensive rejection of drug therapy (or any other specific intervention that we do not happen to provide). Our position on drug use should be precisely the same as medicine: all drug use should be appropriate and guided by the scientific literature. And we should acknowledge that sometimes the correct treatment would involve drugs.

The decision to reject the use of drugs should always be contingent upon the scientific literature. The literature currently provides that conservative and manual therapies are legitimate treatment options for a large percentage of the patient population with spinal complaints. Until such point as it becomes clear that it is not possible to practice EBHC without drugs (and that point may never arrive) chiropractic should remain committed to conservative manual therapies.

3. Chiropractic education and licensing. The model we have proposed does not require any specific change in chiropractic education or licensure to be implemented. In fact, one criterion behind our model is that it reflects how chiropractors are educated, and how they practice. So, we already have concluded that the *de facto *model being taught at chiropractic colleges is that of a back pain specialist (their proclamations of primary care, notwithstanding). We do believe that a more explicit embrace of the Spine Care model would lead to a higher quality of education. We do, of course, hope that chiropractic education improves, particularly with respect to the patient care component of the education. Similarly, the Spine Care model is completely consistent with current state licensing. There will always be disputes and turf wars at the margins of the licensing process, and there are some onerous elements to some state laws, but we do not propose any wholesale revision or alteration of the statutory scope of chiropractic practice.

4. Wellness/prevention as a principle of chiropractic. Nearly all factions of the profession make the claim that chiropractic represents a "wellness" approach to health. Some factions use this term to mean, "We will prevent disease by eliminating subluxations." Others use the term to mean, "We will prevent back pain and related disorders by providing comprehensive spine care." And still others use the term to mean, "We will prevent a variety of degenerative diseases (cardiovascular, neoplastic, etc.) by advising patients on how to live a more healthy life." The first example is unproven and unlikely to be true. The second two examples are also unproven, although they are not scientifically implausible as is the first example. The question is whether the chiropractic can actually deliver on the promise to promote health and prevent disease (as opposed to treating symptomatic patients). To date chiropractic has not demonstrated that it can deliver on the promise of prevention. It is difficult to make the case that chiropractic, uniquely or distinctively among health profession, is concerned with, and capable of providing effective preventive health care. Chiropractors should certainly concern themselves with patients' behavior that may affect patients' health, and provide whatever advice, council, and encouragement they can to improve health related behavior. But, until we can demonstrate that we are effective where others are not, the proposition of chiropractic as the "wellness profession" is not defensible.

## Summary

To date, the chiropractic profession has failed to develop the legitimacy necessary to defend its autonomy and cultural authority. It has not shown the will or ability to define for itself a coherent and consistent identity that takes into account the realities of the healthcare world in which we operate. If the profession fails to do so its future will be imperiled. We offer a professional model for the chiropractic profession. The essential characteristics of this model are:

• Spinal care as the defining clinical purpose of chiropractic.

• Chiropractic as a portal-of-entry provider.

• The acceptance and promotion of EBHC.

• A conservative clinical approach.

• Chiropractic as an integrated part of the healthcare mainstream

• The rigorous implementation of accepted standards of professional ethics.

## Authors' contributions

The cover letter describes the general context and process by which this manuscript was created. After the first set of meetings Dr. Nelson wrote an initial draft document, which reflected the collective thoughts and analyses of the participants. All of the authors participated and contributed in the initial and subsequent meetings during which the overall themes were identified and described. All of the authors made original contributions to the content of the draft and final manuscript. And all of the authors participated in the editing and revisions of the multiple drafts that existed between the initial and final draft.
